# Supplementary material for: Xanthate-Modified Magnetic Fe3O4@SiO2-Based Polyvinyl Alcohol/Chitosan Composite Material for Efficient Removal of Heavy Metal Ions from Water
Source: Polymers (Basel). 2022 Mar 10;14(6):1107. doi: 10.3390/polym14061107 (PMC8951190; doi:10.3390/polym14061107)
Supplement: Supplementary file 1 [file polymers-14-01107-s001.zip › polymers-1585907-supplementary.pdf]

polymers

MDPI

# Supplementary Information

## 1. Experimental

### *1.1. Swelling experiments*

To evaluate the durability of XMPC, deionized water (pH = 7), acidic (pH = 3, aqueous hydrochloric acid), and basic media (pH = 11, aqueous sodium hydroxide) were used for the swelling test. 1 g of sample immersed in 200 mL deionized water, acidic, and basic media for 10 days, respectively. Then the swollen samples were weighted immediately after removing excess water. The swelling ratio (Sw) is calculated as:

$$Sw = (Ws–Wd)/W\ d \quad \text{(S1)}$$

where *Wd* and *Ws* are the masses before and after immersed for 10 days, respectively.

## 2. Results and discussion

### *2.1. Adsorption studies*

The adsorption capacity $Q_e$ (mg/g) of XMPC toward metal ions at equilibrium was calculated as following Equation S1:

$$Q_e = (Co - Ce)V/m \quad \text{(S2)}$$

where $C_0$ and $C_e$ are the initial and equilibrium concentrations (mg/L) of metal ions in solution, respectively. V (L) is the volume of solution and m (g) is the mass of XMPC.

### *2.2. Adsorption kinetics*

Pseudo-first-order model:

$$\lg(Q_e - Q_t) = \log Q_e - k_1 t \quad \text{(S3)}$$

Pseudo-second-order model:

$$\frac{t}{Q_t} = \frac{1}{k_2 Q_e^2} + \frac{1}{Q_e} t \quad \text{(S4)}$$

Intraparticle diffusion model:

$$Q_t = k_3 t^{1/2} + C \quad \text{(S5)}$$

Where $k_1$ (1/min) and $k_2$ g/(mg·min) are the rate constants for first-order and second-order models, respectively; $k_3$ [mg/(g·min$^{1/2}$)] is a constant related to the diffusion coefficient in intraparticle diffusion model; C is the intercept for the intraparticle diffusion model; $Q_e$ is the fitted adsorption value (mg/g) at equilibrium, and $Q_t$ is the experimental value (mg/g) at set time t (min), respectively.

### *2.3. Adsorption isotherms*

Langmuir model:

$$\frac{C_e}{Q_e} = \frac{C_e}{Q_m} + \frac{1}{K_l Q_m} \quad \text{(S6)}$$

Freundlich model:

$$\log Q_e = b_F \log C_e + \log K_F \quad \text{(S7)}$$

Where $Q_m$ is the maximum adsorption capacity (mg/g), $C_e$ is the final equilibrium mercury concentration (mg/L), $K_L$ is the Langmuir constant (L/mg) related to the adsorption strength. $K_F$ is the Freundlich constant related to the adsorption strength (mg/g) (L/mg),

Langmuir isotherm describes a monolayer adsorption which takes place at homogeneous sites within the adsorbent where all the adsorption sites are energetically identical. Freundlich isotherm expresses adsorption at multilayer and on the energetically heterogeneous surface and active sites.

### 2.4. Absorption thermodynamics

$$\Delta G^{\circ} = -RT\ln K_0 \tag{S8}$$

$$\ln K_0 = \frac{\Delta S^{\circ}}{R} - \frac{\Delta H^{\circ}}{RT} \tag{S9}$$

Where $K_0$ is distribution coefficient, R is the gas constant (8.314 J / (mol·K)), T is the temperature (K).$\Delta G_0$ is the standard Gibbs free energy change, ΔH0 is the standard enthalpy change (J/mol), and $\Delta S_0$ is the standard entropy change (J/(mol·K)). $K_0$ was obtained from the intercept of plotting ln(qe/Ce) versus Ce at three different temperatures by extrapolating Ce to zero. $\Delta H_0$ and $\Delta S_0$ were obtained from the slope and intercept in the curve of $\ln K_0$ versus $T^{-1}$.

### 2.5. Selective adsorption behaviors of heavy metals

The selectivity of XMPC for metal ions over other metal ions can be evaluated using the selectivity coefficient (Ks). It is an important parameter to express the removal selectivity of the adsorbent toward objective metal ions in a complex components solution, which can be derived by the following Equation S10:

$$K_s = \frac{K_d(T)}{K_d(I)} \tag{S10}$$

where Kd is the distribution coefficient of metal ions and was calculated by Equation S11:

$$K_d = \frac{Q_e}{C_e} \tag{S11}$$

**Table S1.** Swelling ratio of XMPC.

| pH | $W_d$ (g) | $W_S$ (g) | $S_w$ |
|---|---|---|---|
| 3 | 0.20 | 1.52 | 6.6 |
| 7 | 0.20 | 1.37 | 5.8 |
| 11 | 0.20 | 1.29 | 5.5 |

**Table S2.** Adsorption and desorption data of heavy metal ion adsorption by XMPC.

| Time | Qe(mg/g) | | | Adsorption rate (%) | | | Desorption rate (%) | | |
|---|---|---|---|---|---|---|---|---|---|
| | Pb(II) | Cu(II) | Cd(II) | Pb(II) | Cu(II) | Cd(II) | Pb(II) | Cu(II) | Cd(II) |
| 1 | 62.19 | 89.96 | 116.28 | 33.05 | 54.18 | 86.95 | 89.35 | 81.82 | 84.86 |
| 2 | 54.78 | 84.26 | 100.15 | 27.32 | 49.06 | 66.83 | 79.52 | 79.43 | 70.13 |
| 3 | 48.60 | 78.97 | 97.15 | 24.68 | 44.76 | 41.65 | 62.66 | 72.80 | 63.20 |
| 4 | 42.93 | 81.51 | 81.60 | 22.00 | 43.03 | 48.46 | 60.33 | 53.85 | 61.63 |
| 5 | 40.09 | 60.17 | 77.56 | 18.79 | 35.91 | 44.71 | 59.01 | 57.37 | 49.27 |
| 6 | 40.35 | 55.39. | 64.99 | 18.42 | 34.98 | 34,23 | 55.27 | 47.54 | 45.67 |
| 7 | 36.10 | 55.09 | 54.00 | 17.18 | 31.13 | 26.74 | 41.19 | 41.99 | 42.57 |
| 8 | 26.62 | 84.81 | 34.37 | 12.13 | 25.50 | 19.46 | 38.05 | 34.56 | 41.93 |
| 9 | 26.81 | 49.94 | 26.48 | 12.72 | 26.53 | 14.38 | 40.65 | 35.30 | 41.21 |
| 10 | 24.60 | 41.81 | 37.41 | 10.99 | 20.15 | 16.38 | 39.68 | 42.96 | 30.26 |

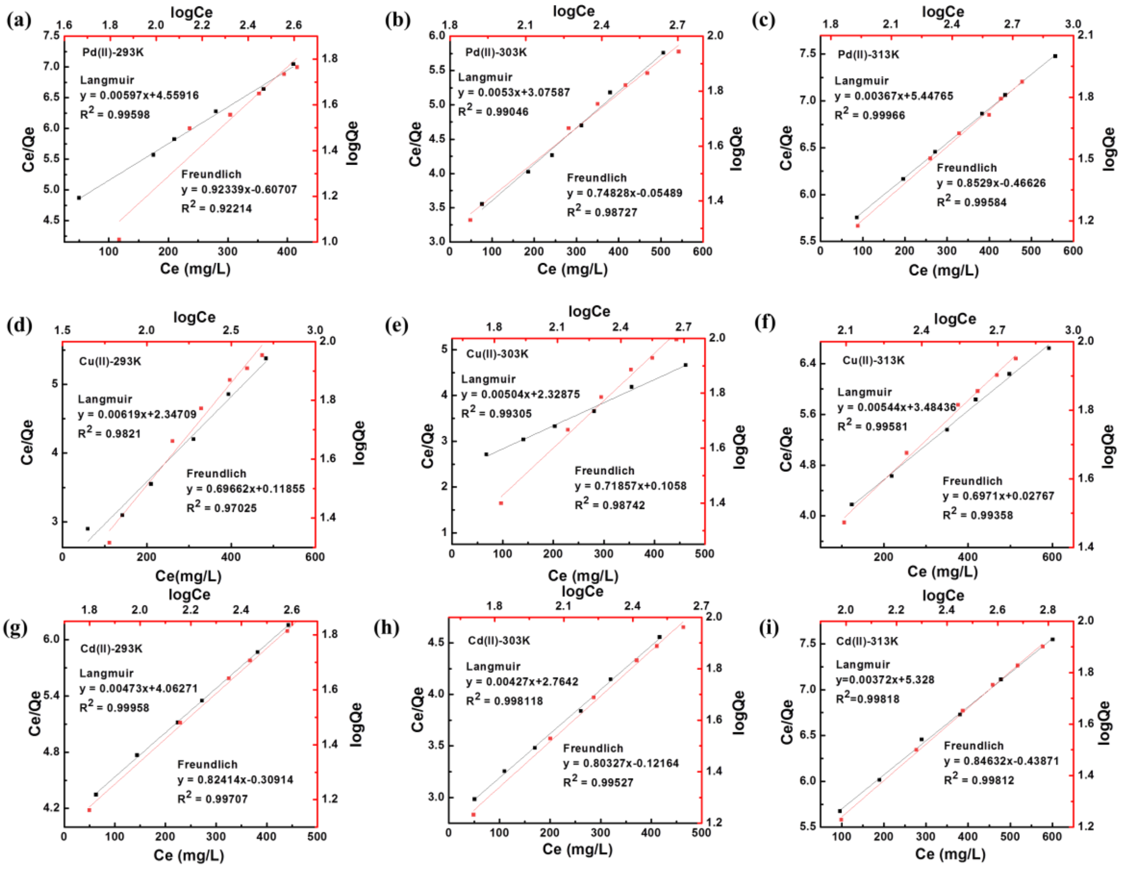


**Figure S1.** Adsorption isotherms for metal ions on XMPC. (**a**) Pd(II) at 293K, (**b**) Pd(II) at 303K, (**c**) Pd(II) at 313K, (**d**) Cu(II) at 293K, (**e**) Cu(II) at 303K, (**f**) Cu(II) at 313K, (**g**) Cd(II) at 293K, (**h**) Cd(II) at 303K, and (**i**) Cd(II) at 313K.
